# Subthreshold Cannabidiol Potentiates Levetiracetam in the Kainic Acid Model of Temporal Lobe Epilepsy: A Pilot Study

**DOI:** 10.3390/ph17091187

**Published:** 2024-09-10

**Authors:** Chiara Lucchi, Mattia Marcucci, Kawther Ameen Muhammed Saeed Aledresi, Anna-Maria Costa, Giuseppe Cannazza, Giuseppe Biagini

**Affiliations:** 1 Department of Biomedical, Metabolic and Neural Sciences, University of Modena and Reggio Emilia, 41125 Modena, Italy; chlucchi@unimore.it (C.L.); 282065@studenti.unimore.it (M.M.); kawtheralidressi1998@gmail.com (K.A.M.S.A.); annamaria.costa@unimore.it (A.-M.C.); 2 Department of Life Sciences, University of Modena and Reggio Emilia, 41125 Modena, Italy; giuseppe.cannazza@unimore.it

**Keywords:** cannabidiol, epilepsy, kainic acid, levetiracetam

## Abstract

Refractoriness to antiseizure medications is still a major concern in the pharmacotherapy of epilepsy. For this reason, we decided to evaluate the combination of levetiracetam and cannabidiol, administered at a subthreshold dose, to limit the possible adverse effects of this phytocannabinoid. We administered levetiracetam (300 mg/kg/day, via osmotic minipumps), cannabidiol (120 mg/kg/day, injected once a day subcutaneously), or their combination for one week in epileptic rats. Saline-treated epileptic rats were the control group. Animals were monitored with video electroencephalography the week before and after the treatment. No changes were found in the controls. Levetiracetam did not significantly reduce the total seizure number or the overall seizure duration. Still, the overall number of seizures (*p* < 0.001, Duncan’s new multiple range test) and their total duration (*p* < 0.01) increased in the week following treatment withdrawal. Cannabidiol did not change seizures when administered as a single drug. Instead, levetiracetam combined with cannabidiol resulted in a significant reduction in the overall number and duration of seizures (*p* < 0.05), when comparing values measured during treatment with both pre- and post-treatment values. These findings depended on changes in convulsive seizures, while non-convulsive seizures were stable. These results suggest that cannabidiol determined a remarkable potentiation of levetiracetam antiseizure effects at a subthreshold dose.

## 1. Introduction

Cannabidiol is an antiseizure medication (ASM) used as add-on drug for some types of pediatric epilepsies, including Dravet syndrome [1,2], Lennox–Gastaut syndrome [3,4], and tuberous sclerosis complex [5], disorders for which the use of cannabidiol has been approved by the U.S. Food and Drug Administration [6]. Although considered effective and safe, cannabidiol is currently not approved for other types of epilepsy in humans. Cannabidiol is also under investigation in veterinary medicine, and one recent trial suggested that this ASM is effective for dogs with idiopathic epilepsy. In these animals, cannabidiol was used as an add-on drug obtaining beneficial results, but at the cost of significant adverse events, mainly consisting of reduced food intake and vomiting [7]. In children with epilepsy, cannabidiol was also associated with adverse events such as diarrhea, increased occurrence of seizures, or sedation, just to mention the more frequent. Reduced appetite and vomiting were also present in the same cohort [8].

The effects of cannabidiol were not investigated in patients with temporal lobe epilepsy (TLE), in spite of the fact that this type of epilepsy is the most prevalent and also the less responsive to ASMs in the population of adults with epileptic disorders [9]. In contrast to the lack of clinical investigations on cannabidiol for TLE, this drug has been studied in various TLE animal models, including kindling in amygdala [10] and hippocampus [6], or the pilocarpine [11] and kainic acid [12] models of status epilepticus. In these previous experiments, and more recently in our rats treated with kainic acid [13], the antiseizure properties of cannabidiol have been consistently documented in animals reproducing human TLE.

The possible interest in clinical use of cannabidiol in TLE is related to the problem of drug refractoriness, which is frequently found in this type of epilepsy [9]. First-line drugs such as levetiracetam were shown to be poorly effective in controlling seizures in as much as 50% of patients with epilepsy [14]. Consistent with these clinical data, levetiracetam administered at 300 mg/kg/day was only partially effective in reducing seizures in epileptic rats that were previously treated with kainic acid [15]. In these rats, levetiracetam produced significant effects mainly on the seizure duration and not on seizure frequency, at least in the animals which were refractory to this ASM (i.e., with persistence of spontaneous recurrent seizures, SRSs). Instead, in the same model, cannabidiol reduced not only the seizure duration, but also the overall seizure occurrence when used at high doses (120 mg/kg bis in die, b.i.d.) [13].

In view of these partially positive findings obtained with levetiracetam in kainic acid-treated epileptic rats, we hypothesized that there could be a potentiation of levetiracetam by combining this ASM with cannabidiol. We also preferred to test a cannabidiol dose lower than that previously used in our experiment [13], so as to evaluate a condition in which the adverse effects of cannabidiol could be less probable. For this reason, and also to simplify the treatment protocol, we performed just one instead of two daily injections of cannabidiol. Here we illustrate an interesting interaction between levetiracetam (administered at 300 mg/kg/day) and cannabidiol, the latter at a dose (120 mg/kg/day) that resulted to be subthreshold in the absence of levetiracetam.

## 2. Results

We considered 26 rats for four groups of treatment (n = 6/7 rats per group). All of them survived to the kainic acid administration (15 mg/kg, intraperitoneally, i.p.), but four of them were non-responders (i.e., they did not develop SRSs). Thus, 22 rats were considered eligible for saline or drug treatment at the end of the monitoring period. After having induced the status epilepticus, epileptic activity was continuously recorded using video electrocorticography (vECoG) until the animals were implanted with osmotic minipumps releasing either saline or levetiracetam [15], with the exception of non-responders which were disregarded. The vECoG recording continued for one week following the treatment period. Subgroups of these rats were also treated with a single daily injection of cannabidiol (120 mg/kg, subcutaneously, s.c.; n = 4, since three additional rats belonging to this group did not develop SRSs) for 7 days, matching the full-time infusion interval by the minipumps. This protocol resulted in four sets of animals (saline released by minipumps, n = 6; levetiracetam (by minipumps), n = 7; saline (by minipumps) + cannabidiol (s.c.), n = 4; cannabidiol (s.c.) + levetiracetam (by minipumps), n = 5, this because one further rat was discarded in this group since it did not develop SRSs). Seizure occurrence and duration were analyzed starting one week before treatment (as previously detailed, four rats were disregarded for treatment, because they did not present SRSs in this week), during treatment for one week, and the following week after treatment. The four rats which did not present SRSs in the first week of monitoring were recorded, but not treated, also in the following two weeks, thus confirming the lack of epileptic activity.

### 2.1. Characterization of the Overall Number of Spontaneous Recurrent Seizures (SRSs)

**Figure 1:**
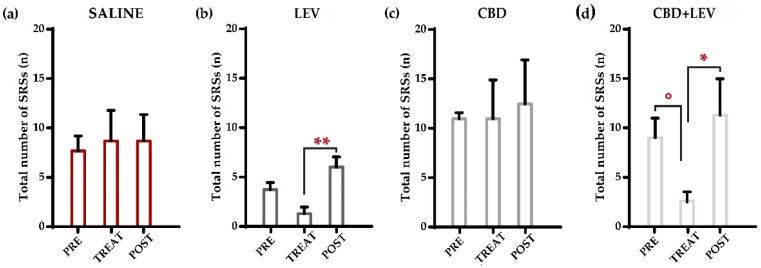
Weekly occurrence of spontaneous recurrent seizures (SRSs) in epileptic rats treated with saline subcutaneously (s.c., via osmotic minipumps [15]) (**a**), saline (s.c.) and levetiracetam (LEV, s.c. via osmotic minipumps [15]) (**b**), saline (s.c., via osmotic minipumps) and cannabidiol (CBD, 120 mg/kg s.c. [13]) (**c**), and cannabidiol + levetiracetam (CBD + LEV; CBD s.c., LEV s.c. via osmotic minipumps) (**d**). In (**a**), the overall number of seizures in rats receiving saline treatment did not change across treatment conditions. In (**b**), after the removal of minipumps, rats treated with levetiracetam presented a significant increase (** *p* < 0.01) in the occurrence of all SRSs. Conversely, cannabidiol treatment (**c**) did not affect the total number of SRSs during the different treatment conditions. In (**d**), the combined treatment of LEV and CBD significantly reduced (° *p* < 0.05) the number of total SRSs in comparison to the pretreatment period. Subsequently, the rapid stop-removal of the treatment led to a significant increase (* *p* < 0.05) in the weekly frequency of SRSs in the post-treatment phase. ** *p* < 0.01, Duncan’s method, TREAT vs. POST in LEV; ° *p* < 0.05 PRE vs. TREAT in CBD + LEV, * *p* < 0.05 TREAT vs. POST in CBD + LEV, Duncan’s new multiple range test.

All rats were nonresponsive to ASMs administered in this study, meaning that SRSs did not disappear during treatment in none of the animals. Figure S1 illustrates the daily changes in SRSs of each rat during the monitoring period. Following the Shapiro–Wilk test, a two-way (treatment × time interval) repeated measures analysis of variance (ANOVA) was performed. As shown in Figure 1a–d, we initially evaluated all the seizures that occurred during the different treatment conditions (i.e., preceding the minipump implantation, during saline or drug delivery for one week (implanted minipumps), and after the removal of minipumps (treatment withdrawal). Total SRSs decreased significantly (*p* < 0.01) within treatment groups. Notably, SRSs showed a significant reduction (−75%) in comparison to the pretreatment period only when levetiracetam was combined with cannabidiol (° *p* < 0.05 according to Duncan’s new multiple range test, MRT). No significant changes were observed in the other treatment groups by comparing pretreatment, treatment, and post-treatment values. However, the comparison of post-treatment and treatment values revealed significant differences in both levetiracetam (** *p* < 0.01) and cannabidiol + levetiracetam (* *p* < 0.05) groups, thus suggesting an effect due to drug withdrawal in both groups receiving the ASMs.

**Figure 2:**
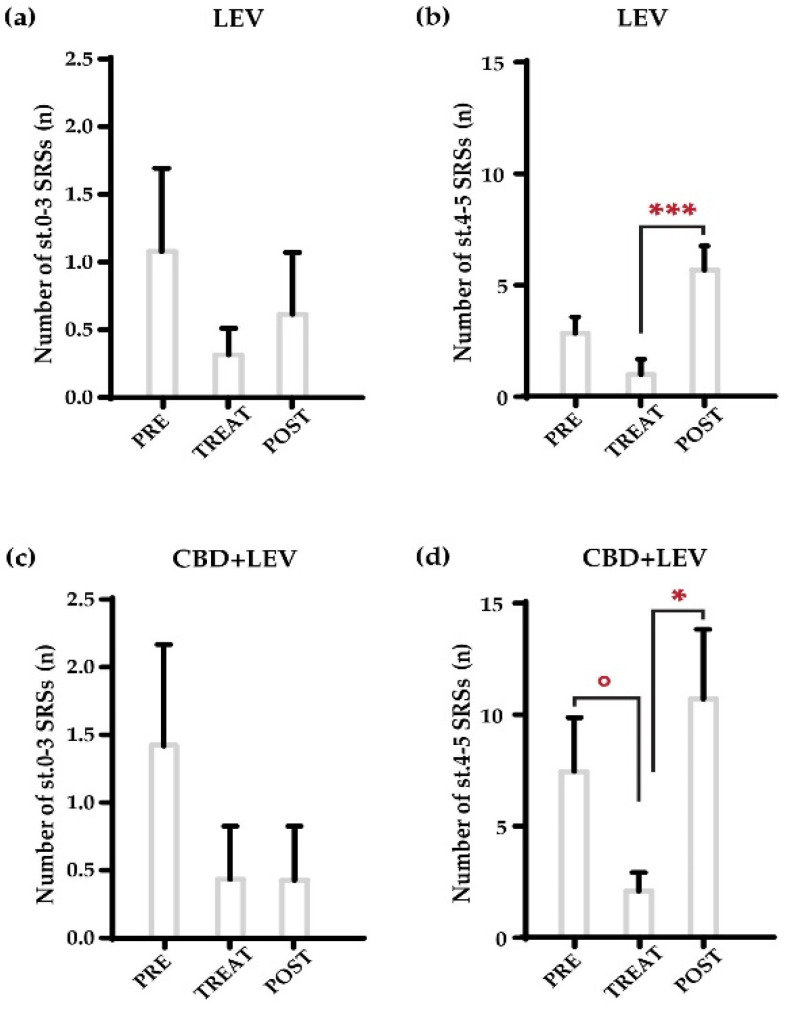
Weekly occurrence of nonconvulsive (stages, st. 0–3 of the Racine’s scale [16]) and convulsive (st. 4–5) spontaneous recurrent seizures (SRSs) in epileptic rats treated with levetiracetam (LEV s.c., via osmotic minipumps) (**a**,**b**) and cannabidiol + levetiracetam (CBD + LEV; CBD s.c., LEV s.c. via osmotic minipumps) (**c**,**d**). As shown in (**a**,**b**), levetiracetam did not significantly reduce the number of both nonconvulsive and convulsive seizures. The removal of minipumps (**b**) resulted in a significant increase (*** *p* < 0.01) in the occurrence of convulsive tonic–clonic seizures in comparison to the treatment period. In (**d**), the combined administration of cannabidiol and levetiracetam significantly affected (° *p* < 0.05) the number of convulsive seizures, in comparison to the pretreatment period. Moreover, the weekly frequency of tonic–clonic SRSs increased significantly (* *p* < 0.05) in the post-treatment phase as a result of the treatment’s quick stop-removal. The number of nonconvulsive seizures remained unchanged. *** *p* < 0.01, Duncan’s new multiple range test (MRT), TREAT vs. POST in LEV; ° *p* < 0.05 PRE vs. TREAT in CBD + LEV, * *p* < 0.05 TREAT vs. POST in CBD + LEV; MRT.

We further analyzed changes in the overall seizure occurrence found in the treatment groups that received levetiracetam and its combination with cannabidiol, by separately analyzing nonconvulsive (stages, st. 0–3 of the Racine’s scale [16], in Figure 2) and convulsive (tonic–clonic, st. 4–5) seizures (Figure 2a–d). The combination treatment primarily affected the number of convulsive seizures (Figure 2d), as the MRT only revealed a significant reduction in the number of tonic–clonic SRSs during drug administration (° *p* < 0.05 vs. pretreatment values in the same group; MRT), followed by the return to previous values after treatment withdrawal (* *p* < 0.05, vs. treatment values in the same group). Additionally, we observed a significant increase in the occurrence of convulsive SRSs after the removal of minipumps loaded with levetiracetam (*** *p* < 0.001, vs. treatment values in the same group), with no changes for non-convulsive SRSs (Figure 2a,b). No changes at all were found for cannabidiol or saline treatments for both nonconvulsive and convulsive seizures.

### 2.2. Characterization of the Total Duration of SRSs

**Figure 3:**
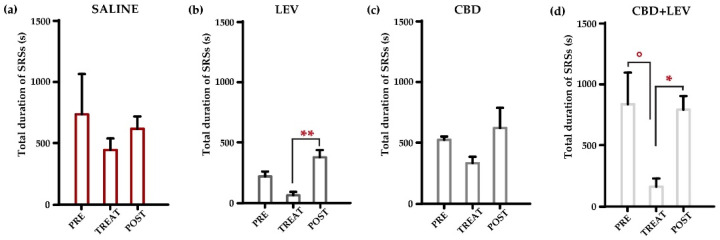
Overall duration of spontaneous recurrent seizures (SRSs) in epileptic rats treated with saline subcutaneously (s.c., via osmotic minipumps [14]) (**a**), saline (s.c.) and levetiracetam (LEV, s.c. via osmotic minipumps [15]) (**b**), saline (s.c., via osmotic minipumps) and cannabidiol (CBD, 120 mg/kg s.c. [12]) (**c**), and cannabidiol + levetiracetam (CBD + LEV; CBD s.c., LEV s.c. via osmotic minipumps) (**d**). In (**a**), note that the total seizure duration in rats receiving saline treatment did not change across treatment conditions. In (**b**), the rapid discontinuation of levetiracetam administration induced a significant increase (** *p* < 0.01) in the overall duration of SRSs, measured during the week. Cannabidiol treatment (**c**) reduced the total seizure duration without reaching a statistical difference. In (**d**), cannabidiol and levetiracetam combination resulted in a beneficial effect on the total duration of SRSs, when compared to pretreatment period (° *p* < 0.05). The cessation of combined treatment subsequently led to a significant increase (* *p* < 0.05) in the overall duration of the SRSs. ** *p* < 0.01, Duncan’s new multiple range test (MRT), TREAT vs. POST in LEV; ° *p* < 0.05 PRE vs. TREAT in CBD + LEV, * *p* < 0.05 TREAT vs. POST in CBD + LEV, MRT.

Visual inspection revealed that the duration of SRSs observed in the week before minipump implantation, and the week following the treatments, differed from that characterized during the treatment period (Figure S2). For this reason, we evaluated the overall duration of epileptic activity in the recorded traces for each group of treatment through a two-way (treatment × time interval) repeated measures ANOVA. This analysis revealed significant variations (*p* < 0.001) in the total duration of SRSs (Figure 3a,d). According to MRT, the SRSs’ overall duration considerably decreased (−82%) during the combined treatment with cannabidiol and levetiracetam (° *p* < 0.05), compared to the pretreatment period (Figure 3d). Then, the prompt cessation of combined treatment led to a significant increase (+520%, * *p* < 0.05) in the overall duration of SRSs (Figure 3d). Additionally, the rapid discontinuation of levetiracetam administration induced an abrupt increase in the overall duration of SRSs, up to 600% of values measured during the treatment (** *p* < 0.01, Figure 3b). In contrast, cannabidiol administration or its withdrawal did not significantly modify the overall seizure duration (Figure 3c). No differences were found in the saline group at all during the considered time intervals (Figure 3a).

### 2.3. Characterization of the Mean Duration of SRSs

**Figure 4:**
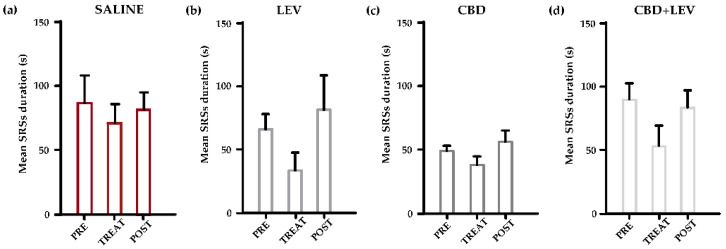
Mean duration of spontaneous recurrent seizures (SRSs) in epileptic rats treated with saline subcutaneously (s.c., via osmotic minipumps) (**a**), saline (s.c.) and levetiracetam (LEV, s.c. via osmotic minipumps [15]) (**b**), saline (s.c., via osmotic minipumps) and cannabidiol (CBD, 120 mg/kg s.c. [13]) (**c**), and cannabidiol + levetiracetam (CBD + LEV; CBD s.c., LEV s.c. via osmotic minipumps) (**d**). A reduction in the mean duration of SRSs was evident in all groups of treatment with minipumps, but the two-way (treatment × time interval) repeated measures ANOVA did not reveal any statistical differences.

To understand if the changes observed in the overall duration of epileptic activity could be related to a reduction in the single seizure duration or, on the contrary, were due to the previously found decrease in the seizure occurrence, we analyzed the mean seizure duration by the repeated measures two-way ANOVA, which did not reveal significant differences among the considered groups of treatment (Figure 4a–d).

## 3. Discussion

In this study we found that: (i) cannabidiol is ineffective when administered once in a day in rats developing epilepsy after a status epilepticus induced by i.p. injecting kainic acid, even when a high dose is used. This finding contrasts sharply with our previous study, which showed a beneficial effect of cannabidiol administered b.i.d. at 120 mg/kg [13]; (ii) although cannabidiol was administered at a subthreshold dose, it potentiated the effects of levetiracetam, which was ineffective when administered alone in epileptic rats. This finding is of interest because it shows that a second-line ASM can be therapeutically useful even at a subthreshold dose, when combined with a first-line ASM (levetiracetam) toward which refractoriness is present. If confirmed by further experiments, this result might suggest that cannabidiol can be used at a dose able to reduce the occurrence of most serious adverse events of this drug.

This observation raises the question of the possible basis for the potentiation of levetiracetam by cannabidiol. While we do not have an answer, a suggestive hypothesis could be based on our previous results [15]. We previously noticed that levetiracetam significantly increases brain levels of allopregnanolone in epileptic rats, especially in the neocortex. Allopregnanolone is the major representative of a class of molecules known as neurosteroids that act as modulators of neuronal excitability [17]. Interestingly, allopregnanolone has been recognized to be an anticonvulsant in TLE [18] and led to development of an ASM, ganaxolone, presently used for seizures associated with the cyclin-dependent kinase-like 5 deficiency disorder [19,20,21].

Since cannabidiol can interact with a variety of targets in the brain [22], multiple mechanisms could be involved in the hypothesized synergism of cannabidiol and levetiracetam-induced allopregnanolone upregulation. Recently, cannabidiol was found to modulate γ-aminobutyric type A (GABA_A_) receptor-mediated currents [23] generated in *Xenopus laevis* oocytes, transplanted with GABA_A_ receptors obtained from the neocortex of patients with TLE. GABA_A_ receptors are well-known targets for allopregnanolone, which is able to positively modulate the GABA_A_-mediated inhibitory currents [24], thus reducing neuronal excitation in the brain. Interestingly, cannabidiol in the low micromolar range mainly modifies currents produced by α1-6βγ2 GABA_A_ receptors. The α2-containing GABA_A_ receptors appear to be more responsive to cannabidiol, which is able to produce a four-fold increase of their currents. A β-subunit selectivity was also reported by the same investigators, with a prevalence of β2/β3 over β1 subunits [22]. Several binding sites have been identified in the GABA_A_ receptor for allopregnanolone, both in α and β subunits [25], suggesting the possibility that the modulation of these GABA_A_ receptor components could underpin the reported synergism between cannabidiol and the allopregnanolone analog ganaxolone [26].

Other possible factors can be considered to explain the potentiation of levetiracetam effects by subthreshold cannabidiol. Cannabidiol was reported to alter kinetics and dynamics of various ASMs, including valproate, clobazam, and levetiracetam [27]. Specifically, in the case of levetiracetam a reduction in the antiseizure effects of this ASM was evidenced in presence of cannabidiol, when both these drugs were tested in mice with 6 Hz corneally induced seizures [28]. However, this finding was the opposite of that observed in our animals. Moreover, in a clinical study based on children and adult patients with epilepsy and treated with various ASMs, levetiracetam serum levels were increased by cannabidiol administered as an add-on drug, but only in the presence of pediatric epilepsy [29]. Although we could not exclude a similar phenomenon in our adult rats, we believe that it was improbable.

In our rats, we observed an incomplete response to the combined treatment, which means that levetiracetam and cannabidiol were able to significantly reduce the occurrence and severity of seizures, without abolishing them, a phenomenon well described in human patients [30]. Levetiracetam, instead, was not able to significantly modify symptoms of epilepsy when administered as single ASM, at least in our rats. This observation was consistent with other experiments in which both female [31] and male [32] mice, or male rats [33] developed levetiracetam refractory seizures after kainic acid administration, at a dose of this ASM comparable to that used in our rats. At variance, in a previous study we reported that approximately half of male rats completely responded to levetiracetam with seizure suppression, whereas the others did not respond, as in the present study [15]. Interestingly, in non-responders we observed a rebound effect due to levetiracetam withdrawal, consisting of increased seizure occurrence reaching a statistically significant level, as in the present experiment.

Other investigators found that much higher levetiracetam doses (up to 800 mg/kg) were effective in suppressing seizure in the intrahippocampal kainic acid model of TLE, in mice. The same investigators observed a large inter-individual variability in the response to levetiracetam [34], as also observed in our rats [15]. Unfortunately, variability in the response to levetiracetam in rodent models of epilepsy, and human patients [30], is a recognized problem that could not be ruled out even by increasing the number of investigated subjects, as we did in our previous study based on 15 levetiracetam-treated rats [15]. However, it is interesting to note that the combination of levetiracetam and subthreshold cannabidiol resulted in statistically significant beneficial results, in spite of the low number of considered epileptic rats. The therapeutic effects were on the number of seizures, whereas no significant changes concerned the mean seizure duration. Also in our previous experiment [15], the effects of levetiracetam on the seizure duration were inconsistent in the course of the treatment week, with only 3 days in which a reduction was statistically confirmed. Instead, cannabidiol was effective in reducing the seizure duration when administered b.i.d. at 120 mg/kg, but failed in the present experiment because administered at a single dose, even when combined with levetiracetam. This indicates that only certain features of epilepsy could be addressed by the tested levetiracetam and cannabidiol combination.

## 4. Materials and Methods

### 4.1. Animals

The study protocol was authorized by the Italian Ministry of Health (729/2021-PR), after approval by the university Animal Welfare Body. All experiments were performed by the European Directive 2010/63/EU and the consequent Italian act (DM 26/2014). Twenty-six adult Sprague-Dawley male rats (Charles River, Calco, Italy) were housed in a pathogen-free facility with a controlled environment and unlimited access to food and water. A total of 26 rats, with an initial weight of 175–200 g, were used in this study. Every effort was made to refine procedures, improve animal welfare, and minimize the number of animals utilized in experiments.

### 4.2. Experimental Design

Status epilepticus was induced by i.p. injecting kainic acid (15 mg/kg, in saline; Cayman Chemical, Ann Arbor, MI, USA) one week after electrode implantation. To minimize discomfort caused by the status epilepticus, s.c. injection of Ringer’s lactate solution (3–5 mL) along with softened rat chow was administered. Six weeks after kainic acid administration, rats were subjected to the following treatments: (i) saline (n = 6), (ii) levetiracetam (Cayman Chemical, Ann Arbor, MI, USA) (n = 7), (iii) cannabidiol (obtained by synthesis, CAS number: 13956-29-1; Farmabios, Gropello Cairoli, Italy) (n = 4), and (iv) cannabidiol + levetiracetam (n = 5). Four additional rats were non-responders. All rats were anesthetized and implanted subcutaneously with a minipump delivering continuous dosing over one week (2ML1 ALZET, flow rate: 10 µL/h, DURECT Corporation, Cupertino, CA, USA) of saline (i, iii) or levetiracetam, at 300 mg/kg/day [15] (ii, iv). Since this study aimed to evaluate the combination of levetiracetam with a single dose of cannabidiol, group (iv) was treated at the same time with cannabidiol (120 mg/kg, s.c.) dissolved in MCT oil (USP pharmaceutical grade MCT Lean; provided by MCT Foods, Glencoe, IL, USA). The last group (iii) consisted of epileptic rats (n = 4) treated with cannabidiol dissolved in MCT oil (120 mg/kg, s.c) once a day for 7 days, and implanted with a minipump loaded with saline. In all animals, SRSs were continuously video ECoG monitored.

### 4.3. Electrode Implantation and Video-Electrocorticography Analysis

As previously described [13,15], rats were implanted with epidural electrodes in the frontal (bregma 0 mm, 3.5 mm lateral from midline) and occipital cortices (bregma −6.5 mm, 3.5 mm lateral from midline). One electrode was implanted below the lambda in the midline and used as a reference. The ECoG data were recorded via a cable connection between the headset and preamplifiers. Electrical activity was digitally filtered (0.3 Hz high-pass, 500 Hz low-pass) and acquired a 1 kHz per channel. All data were stored on a personal computer after the mathematical subtraction of traces of recording electrodes from the trace of reference electrodes by using a PowerLab8/30 amplifier connected to 4 BioAmp preamplifiers (ADInstruments; Dunedin, Otago, New Zealand). Videos were digitally recorded through a camera connected to the dedicated computer and synchronized to the ECoG traces through the LabChart 8 PRO internal trigger.

Using LabChart 8 PRO (ADInstruments), offline ECoG signals were digitally filtered (band-pass: high, 50 Hz; low, 1 Hz) and manually examined. The ECoG traces were used to identify seizures, and the synchronized video recordings were used to evaluate the animal behaviors. As regards seizures, they were scored as follows: stage 0 if a clear epileptiform ECoG signal was observed without behavioral changes in the video; stage 1–2 in the presence of absence-like immobility, “wet-dog shakes”, facial automatisms, and head nodding; stage 3 when presenting with forelimb clonus and lordosis; stage 4 corresponding to generalized seizures and rearing; and stage 5 when seizures consisted of rearing with the loss of posture and/or wild running, followed by generalized convulsions [16].

### 4.4. Statistical Analysis

We compared the data for all groups using repeated-measures two-way ANOVA, followed by MRT. Specifically, after having tested the normality distribution (Shapiro–Wilk), data on the total number and the duration of SRSs were analyzed by considering treatments and time intervals as the main factors. To evaluate the differences in the type of seizure (nonconvulsive or convulsive), a three-way analysis of variance considered as factors treatments, time intervals, and the seizure type, followed by the MRT. All statistical analyses were carried out using SigmaPlot 11 (Systat Software, San Jose, CA, USA). Data are presented as mean ± standard error of the mean (SEM) and were regarded as significantly different at *p* < 0.05.

## 5. Conclusions

In epileptic rats, we unexpectedly found that cannabidiol can be used as a second-line ASM, at a dose not able to exert any effect without the concomitant administration of the first-line ASM levetiracetam. This discovery could be useful to make the cannabidiol administration more acceptable. However, not all the antiseizure properties of cannabidiol were preserved using this ASM at a subthreshold dose, thus making the decision to use a single administration of cannabidiol more questionable. In any case, our findings open the possibility to use cannabidiol in a more personalized way, so as to limit the sedative effect of this drug and all the other possible adverse events due to its administration at full doses.

## Data Availability

The data is contained within article.

## Supplementary Materials

The following supporting information can be downloaded at: https://www.mdpi.com/article/10.3390/ph17091187/s1, Figure S1: Daily occurrence of spontaneous recurrent seizures (SRSs) in epileptic rats treated with saline subcutaneously (s.c., via osmotic minipumps [15]); Figure S2: SRSs after induction of status epilepticus in epileptic rats treated with saline s.c. (via osmotic minipumps [15]), saline (s.c.) and levetiracetam (LEV, s.c. via osmotic minipumps [15]), saline (s.c., via osmotic minipumps) and cannabidiol (CBD, 120 mg/kg s.c. [13]), and cannabidiol + levetiracetam (CBD+LEV; CBD s.c., LEV s.c. via osmotic minipumps).

## References

[1] Devinsky O., Cross J.H., Laux L., Marsh E., Miller I., Nabbout R., Scheffer I.E., Thiele E.A., Wright S. (2017). Trial of Cannabidiol for Drug-Resistant Seizures in the Dravet Syndrome. N. Engl. J. Med..

[2] Miller I., Scheffer I.E., Gunning B., Sanchez-Carpintero R., Gil-Nagel A., Perry M.S., Saneto R.P., Checketts D., Dunayevich E., Knappertz V. (2020). Dose-Ranging Effect of Adjunctive Oral Cannabidiol vs Placebo on Convulsive Seizure Frequency in Dravet Syndrome: A Randomized Clinical Trial. JAMA Neurol..

[3] Devinsky O., Patel A.D., Cross J.H., Villanueva V., Wirrell E.C., Privitera M., Greenwood S.M., Roberts C., Checketts D., VanLandingham K.E. (2018). Effect of Cannabidiol on Drop Seizures in the Lennox–Gastaut Syndrome. N. Engl. J. Med..

[4] Thiele E.A., Marsh E.D., French J.A., Mazurkiewicz-Beldzinska M., Benbadis S.R., Joshi C., Lyons P.D., Taylor A., Roberts C., Sommerville K. (2018). Cannabidiol in Patients with Seizures Associated with Lennox-Gastaut Syndrome (GWPCARE4): A Randomised, Double-Blind, Placebo-Controlled Phase 3 Trial. Lancet.

[5] Thiele E.A., Bebin E.M., Bhathal H., Jansen F.E., Kotulska K., Lawson J.A., O’Callaghan F.J., Wong M., Sahebkar F., Checketts D. (2021). Add-on Cannabidiol Treatment for Drug-Resistant Seizures in Tuberous Sclerosis Complex: A Placebo-Controlled Randomized Clinical Trial. JAMA Neurol..

[6] Reddy S.D. (2023). Therapeutic and clinical foundations of cannabidiol therapy for difficult-to-treat seizures in children and adults with refractory epilepsies. Exp. Neurol..

[7] Rozental A.J., Weisbeck B.G., Corsato Alvarenga I., Gustafson D.L., Kusick B.R., Rao S., Bartner L.R., McGrath S. (2023). The Efficacy and Safety of Cannabidiol as Adjunct Treatment for Drug-resistant Idiopathic Epilepsy in 51 Dogs: A Double-blinded Crossover Study. J. Vet. Intern. Med..

[8] Szaflarski J.P., Devinsky O., Lopez M., Park Y.D., Zentil P.P., Patel A.D., Thiele E.A., Wechsler R.T., Checketts D., Sahebkar F. (2023). Long-term Efficacy and Safety of Cannabidiol in Patients with Treatment-resistant Epilepsies: Four-year Results from the Expanded Access Program. Epilepsia.

[9] Téllez-Zenteno J.F., Hernández-Ronquillo L. (2012). A Review of the Epidemiology of Temporal Lobe Epilepsy. Epilepsy Res. Treat..

[10] Fallah M.S., Dlugosz L., Scott B.W., Thompson M.D., Burnham W.M. (2021). Antiseizure Effects of the Cannabinoids in the Amygdala-kindling Model. Epilepsia.

[11] Patra P.H., Barker-Haliski M., White H.S., Whalley B.J., Glyn S., Sandhu H., Jones N., Bazelot M., Williams C.M., McNeish A.J. (2019). Cannabidiol Reduces Seizures and Associated Behavioral Comorbidities in a Range of Animal Seizure and Epilepsy Models. Epilepsia.

[12] Thomson K.E., Metcalf C.S., Newell T.G., Huff J., Edwards S.F., West P.J., Wilcox K.S. (2020). Evaluation of Subchronic Administration of Antiseizure Drugs in Spontaneously Seizing Rats. Epilepsia.

[13] Costa A.-M., Russo F., Senn L., Ibatici D., Cannazza G., Biagini G. (2022). Antiseizure Effects of Cannabidiol Leading to Increased Peroxisome Proliferator-Activated Receptor Gamma Levels in the Hippocampal CA3 Subfield of Epileptic Rats. Pharmaceuticals.

[14] Lynch J.M., Tate S.K., Kinirons P., Weale M.E., Cavalleri G.L., Depondt C., Murphy K., O’Rourke D., Doherty C.P., Shianna K.V. (2009). No Major Role of Common SV2A Variation for Predisposition or Levetiracetam Response in Epilepsy. Epilepsy Res..

[15] Costa A.-M., Lucchi C., Malkoç A., Rustichelli C., Biagini G. (2021). Relationship between Delta Rhythm, Seizure Occurrence and Allopregnanolone Hippocampal Levels in Epileptic Rats Exposed to the Rebound Effect. Pharmaceuticals.

[16] Racine R.J. (1972). Modification of Seizure Activity by Electrical Stimulation: II. Motor Seizure. Electroencephalogr. Clin. Neurophysiol..

[17] Biagini G., Panuccio G., Avoli M. (2010). Neurosteroids and Epilepsy. Curr. Opin. Neurol..

[18] Lévesque M., Biagini G., Avoli M. (2020). Neurosteroids and Focal Epileptic Disorders. Int. J. Mol. Sci..

[19] Reddy D.S. (2024). Neurosteroids as Novel Anticonvulsants for Refractory Status Epilepticus and Medical Countermeasures for Nerve Agents: A 15-Year Journey to Bring Ganaxolone from Bench to Clinic. J. Pharmacol. Exp. Ther..

[20] Knight E.M.P., Amin S., Bahi-Buisson N., Benke T.A., Cross J.H., Demarest S.T., Olson H.E., Specchio N., Fleming T.R., Aimetti A.A. (2022). Safety and Efficacy of Ganaxolone in Patients with CDKL5 Deficiency Disorder: Results from the Double-Blind Phase of a Randomised, Placebo-Controlled, Phase 3 Trial. Lancet Neurol..

[21] Olson H.E., Amin S., Bahi-Buisson N., Devinsky O., Marsh E.D., Pestana-Knight E., Rajaraman R.R., Aimetti A.A., Rybak E., Kong F. (2024). Long-term Treatment with Ganaxolone for Seizures Associated with Cyclin-dependent Kinase-like 5 Deficiency Disorder: Two-year Open-label Extension Follow-up. Epilepsia.

[22] Bakas T., Van Nieuwenhuijzen P.S., Devenish S.O., McGregor I.S., Arnold J.C., Chebib M. (2017). The Direct Actions of Cannabidiol and 2-Arachidonoyl Glycerol at GABA_A_ Receptors. Pharmacol. Res..

[23] Ruffolo G., Gaeta A., Cannata B., Pinzaglia C., Aronica E., Morano A., Cifelli P., Palma E. (2022). GABAergic Neurotransmission in Human Tissues Is Modulated by Cannabidiol. Life.

[24] Gunn B.G., Cunningham L., Mitchell S.G., Swinny J.D., Lambert J.J., Belelli D. (2015). GABA_A_ Receptor-Acting Neurosteroids: A Role in the Development and Regulation of the Stress Response. Front. Neuroendocrinol..

[25] Chintala S.M., Tateiwa H., Qian M., Xu Y., Amtashar F., Chen Z., Kirkpatrick C.C., Bracamontes J., Germann A.L., Akk G. (2024). Direct Measurements of Neurosteroid Binding to Specific Sites on GABA_A_ Receptors. Br. J. Pharmacol..

[26] Golub V., Ramakrishnan S., Reddy D.S. (2023). Isobolographic Analysis of Adjunct Antiseizure Activity of the FDA-Approved Cannabidiol with Neurosteroids and Benzodiazepines in Adult Refractory Focal Onset Epilepsy. Exp. Neurol..

[27] Gilmartin C.G.S., Dowd Z., Parker A.P.J., Harijan P. (2021). Interaction of cannabidiol with other antiseizure medications: A narrative review. Seizure.

[28] Socała K., Wyska E., Szafarz M., Nieoczym D., Wlaź P. (2019). Acute effect of cannabidiol on the activity of various novel antiepileptic drugs in the maximal electroshock- and 6 Hz-induced seizures in mice: Pharmacodynamic and pharmacokinetic studies. Neuropharmacology.

[29] Gaston T.E., Bebin E.M., Cutter G.R., Grayson L., Szaflarski J.P. (2023). Final analysis of potential drug-drug interactions between highly purified cannabidiol and anti-seizure medications in an open-label expanded access program. Epilepsia Open.

[30] Bertram E.H., Dudek F.E. (2024). Addressing the problems of treatment failure in epilepsy: You cannot fix what you do not understand. Epilepsia.

[31] Widmann M., Lieb A., Fogli B., Steck A., Mutti A., Schwarzer C. (2024). Characterization of the intrahippocampal kainic acid model in female mice with a special focus on seizure suppression by antiseizure medications. Exp. Neurol..

[32] Duveau V., Pouyatos B., Bressand K., Bouyssières C., Chabrol T., Roche Y., Depaulis A., Roucard C. (2016). Differential Effects of Antiepileptic Drugs on Focal Seizures in the Intrahippocampal Kainate Mouse Model of Mesial Temporal Lobe Epilepsy. CNS Neurosci. Ther..

[33] Casillas-Espinosa P.M., Anderson A., Harutyunyan A., Li C., Lee J., Braine E.L., Brady R.D., Sun M., Huang C., Barlow C.K. (2023). Disease-modifying effects of sodium selenate in a model of drug-resistant, temporal lobe epilepsy. eLife.

[34] Klein S., Bankstahl M., Löscher W. (2015). Inter-individual variation in the effect of antiepileptic drugs in the intrahippocampal kainate model of mesial temporal lobe epilepsy in mice. Neuropharmacology.

