# Supplementary material for: Subthreshold Cannabidiol Potentiates Levetiracetam in the Kainic Acid Model of Temporal Lobe Epilepsy: A Pilot Study
Source: Pharmaceuticals (Basel). 2024 Sep 10;17(9):1187. doi: 10.3390/ph17091187 (PMC11435403; doi:10.3390/ph17091187)

Figure S1. Daily occurrence of spontaneous recurrent seizures (SRSs) in epileptic rats treated with saline subcutaneously (s.c., via osmotic minipumps [15]) (a), saline (s.c.) and levetiracetam (LEV, s.c. via osmotic minipumps [15]) (b), saline (s.c., via osmotic minipumps) and cannabidiol (CBD, 120 mg/kg s.c. [13]) (c), and cannabidiol + levetiracetam (CBD+LEV; CBD s.c., LEV s.c. via osmotic minipumps) (d). Each point represents the number of totals SRSs occurred per day preceding the minipump implantation (PRE), during saline or drug delivery (TREAT), and after the removal of minipumps (POST).

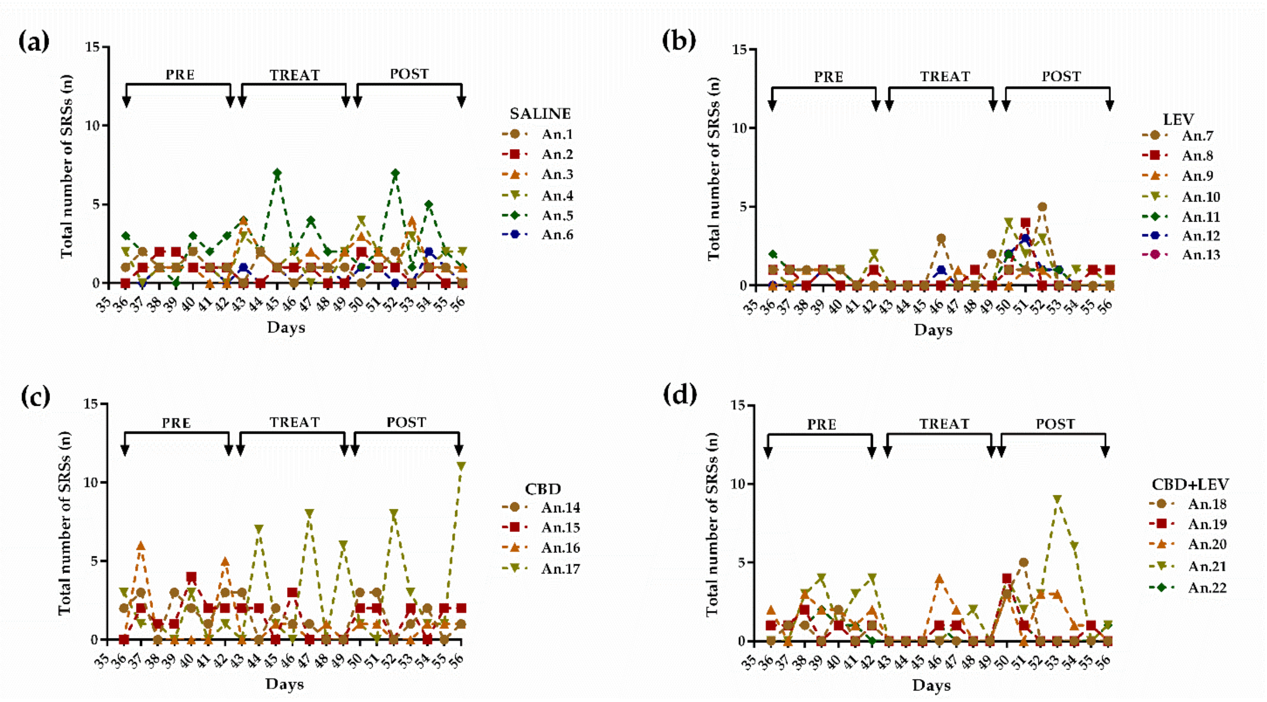

Figure S2. Spontaneous recurrent seizures (SRSs) after induction of status epilepticus in epileptic rats treated with saline subcutaneously (s.c., via osmotic minipumps [15]), saline (s.c.) and levetiracetam (LEV, s.c. via osmotic minipumps [15]), saline (s.c., via osmotic minipumps) and cannabidiol (CBD, 120 mg/kg s.c. [13]), and cannabidiol + levetiracetam (CBD+LEV; CBD s.c., LEV s.c. via osmotic minipumps). In (b) the combined treatment (CBD+LEV) significantly reduced the SRS duration, when compared to the pretreatment period (a). During the post-treatment phase (c), the cessation of the combined treatment led to an increase in the SRS duration. The ECoG traces were obtained from the left frontal (LF) electrodes (d). RF: right frontal, RO: right occipital, Ref: reference, A1: anchor 1, A2: anchor 2. Arrows represent the onset and termination of SRSs.

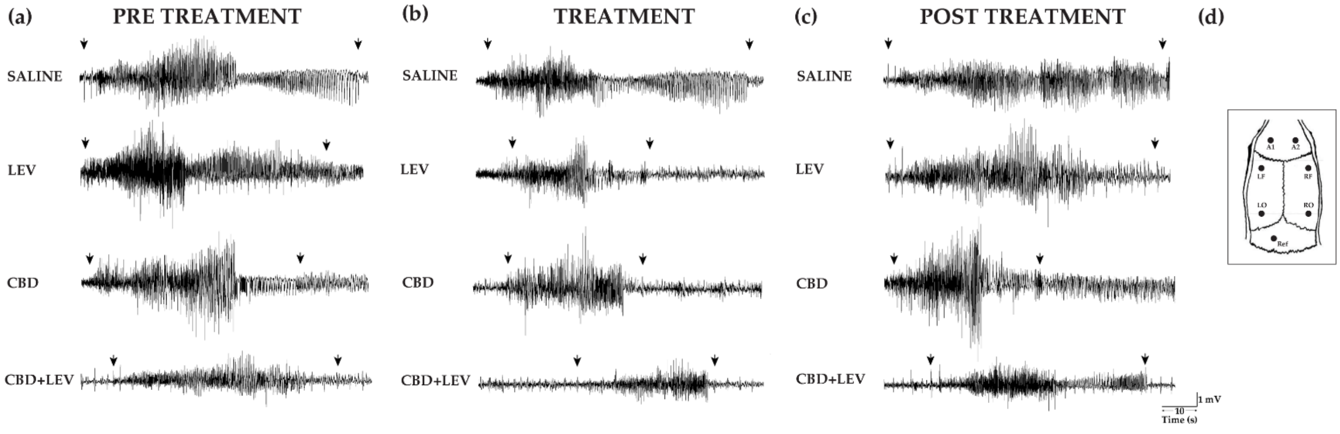

Supplement: Supplementary file 1 [file pharmaceuticals-17-01187-s001.zip › pharmaceuticals-3166140-supplementary.pdf]
